# Association between weight-adjusted-waist index and anxiety among adults in the National Health and Nutrition Examination Survey (NHANES), 2007–2012

**DOI:** 10.3389/fnut.2025.1530028

**Published:** 2025-06-13

**Authors:** Xiao Liang, Kai Chen, Yongcan Xia, Siyuan Ding, Hao Wu, Lijuan Huang, Zhenlin Chen, Yuqian Yan

**Affiliations:** ^1^Department of Oncology, the Affiliated Jiangyin Hospital of Nantong University, Jiangyin, China; ^2^Department of Radiotherapy, The First People’s Hospital of Yancheng, Yancheng, China; ^3^School of Rehabilitation Medicine, Nanjing Medical University, Nanjing, China; ^4^Medical Department, Taizhou Fifth People’s Hospital, Taizhou, China; ^5^Chongqing Jiangbei Second People’s Hospital (Chongqing Jiangbei Mental Health Center), Chongqing, China

**Keywords:** anxiety, weight-adjusted-waist index, NHANES, body mass index (BMI), waist circumference

## Abstract

**Background and aims:**

Anxiety is a pervasive mental health concern and has evolved into a multifaceted and pressing global health concern. Despite this, the connection between the weight-adjusted waist index (WWI) and anxiety symptoms remains unexplored. Therefore, the objective of this study was to evaluate the relationship between anxiety symptoms and WWI.

**Methods:**

Participants were recruited from the National Health and Nutrition Examination Survey (NHANES) spanning the years 2007 to 2012. Anxiety was evaluated based on the patients’ self-reported number of anxious days per month. WWI was calculated by dividing the waist circumference (measured in centimeters) by the square root of the weight (measured in kilograms). To examine linear and non-linear associations between the WWI and anxiety, we employed survey-weighted multivariable logistic regression and generalized additive models. Subgroup analyses were also conducted.

**Results:**

This study comprised 14,677 participants, with 3,745 of them experiencing a state of anxiety. WWI exhibited a positive correlation with anxiety, as evidenced by a fully adjusted odds ratio of 1.11. Upon converting WWI into a categorical variable based on quartiles, participants in the highest quartile had a significantly elevated risk of anxiety compared to those in the lowest quartile. Furthermore, subgroup analyses indicated that the link between WWI and anxiety was more robust among individuals who identified as female, were under 50 years old, were non-Hispanic Black, were separated, were non-smokers, and were non-heavy drinkers.

**Conclusion:**

This study uncovered a notable positive correlation between WWI and anxiety, warranting further validation through future research endeavors.

## 1 Introduction

Anxiety, a pervasive mental health concern, has garnered significant attention due to its substantial contribution to the global disability burden (1). Characterized by intense, persistent feelings of worry and fear, anxiety profoundly affects individuals’ quality of life and overall functioning (2, 3). Based on a meta-analysis, the worldwide incidence rate of anxiety stood at 14.7% (4). Widespread and often accompanied by emotional instability, cognitive difficulties, and social challenges, anxiety disorders underscore the need for a comprehensive understanding of their triggers and exacerbating factors to facilitate the development of effective preventive and therapeutic approaches (3, 5).

Obesity refers to an excessive or abnormal accumulation of body fat, which adversely affects health (6). The prevalence rate of obesity among American adults has climbed to 39.5% and continues to rise (7). Obesity ranks as the sixth most significant risk factor contributing to the global burden of illness (8). Obesity not only correlates with various physiological outcomes but also exhibits a robust association with the onset of chronic diseases and an elevated risk for multiple mental disorders (9–11). Over the past several decades, both body mass index (BMI) and waist circumference (WC) have served as widely-applied conventional indicators for assessing obesity in epidemiological research. However, simple obesity parameters, including BMI and WC, have inherent limitations as they fail to differentiate between fat and muscle weight, thus unable to precisely depict the overall fat content and distribution of abdominal fat (12, 13). Hence, there is a need for a more practical index that takes into consideration these factors and accurately depicts the severity of obesity. The weight-adjusted-waist index (WWI), a pioneering anthropometric measure that normalizes WC relative to body weight, exhibits robust correlations with cardiovascular incidents, fatty liver afflictions, and diabetes (14–17). Moreover, a recent investigation has revealed that WWI serves as a comprehensive index, encapsulating the status of fat, muscle health, and bone mass (18). Numerous studies have demonstrated that WWI exhibits greater accuracy when compared to BMI (19, 20).

However, despite extensive research, there has been no prior investigation exploring the relationship between WWI and anxiety. Consequently, we conducted a cross-sectional study utilizing data from the National Health and Nutrition Examination Survey (NHANES) spanning from 2007 to 2012, in order to delve into this association between WWI and anxiety.

## 2 Materials and methods

### 2.1 Study design and population

The data analyzed in this study was gathered during the 2007–2012 cycles of NHANES, an ongoing series of cross-sectional surveys conducted by the National Center for Health Statistics (NCHS). The NHANES aims to evaluate the health and nutritional status of both adults and children in the United States. To ensure a nationally representative sample of the non-institutionalized US civilian population, the NCHS employs stratified, clustered, multi-stage probability surveys (21, 22). Demographic, socioeconomic status, dietary, lifestyle, and medical condition information for NHANES study participants was gathered by well-trained professionals during in-home interviews. Furthermore, comprehensive physical examinations were conducted, and laboratory tests, including assessments of nutritional status, health, and environmental exposures, were performed at mobile examination centers (23). The program is conducted on a continuous, annual basis and the data is released in a 2-year cycle (24). The study received approval from the Institutional Review Board of the NCHS, and all participants provided written informed consent.

**FIGURE 1:**
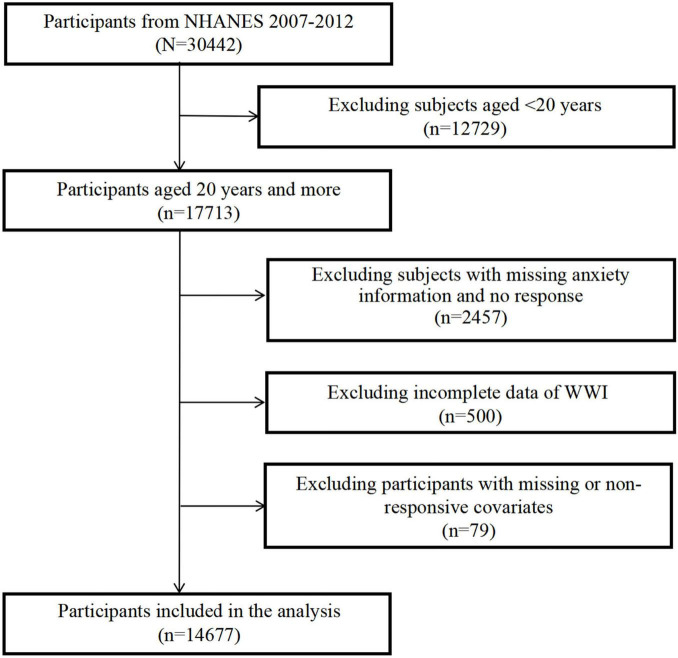
Flow chart for participants recruitment of this study, NHANES 2007–2012.

For this study, data was collected over three 2-year cycles spanning from 2007 to 2012, encompassing a total of 30442 individuals. As a result, 14,677 subjects were included in the analysis. Figure 1 outlines the comprehensive process of sample exclusion. Specifically, we excluded 12,729 participants who were younger than 20 years old, 2,457 participants with missing anxiety information and no response, 500 participants with incomplete data of WWI, and 79 participants with missing or non-responsive covariates.

### 2.2 Definition of anxiety

During the NHANES 2007–2012 study, information on anxiety state was collected through questionnaires administered via a Computer-Assisted Personal Interviewing (CAPI) system at the Mobile Examination Center (MEC) during physical examinations. Participants were asked, “During the past 30 days, for about how many days have you felt worried, tense, or anxious?” to assess their anxiety state. This assessment was based on the 14-item Healthy Days Measures established by the CDC (25), which has been incorporated into health-related quality-of-life (HRQoL) assessments. The reliability of these surveillance questions on HRQoL has been found to be moderate to excellent (26). Consistent with previous studies, anxiety state was categorized as either no (felt anxious for 0–6 days per month) or yes (felt anxious for 7–30 days per month) (27, 28). Additionally, the frequency of anxiety, represented as the number of days (0–30 days per month), was also used as a continuous variable in the analysis of study outcomes. Furthermore, the use of anxiolytics was included as an additional outcome measure, with an anxiolytic state defined as participants experiencing anxiety for six or more days per month or taking one or more anxiolytic medications. The codes and names of drugs used to define the anxiolytic state are provided in Supplementary Table 1.

### 2.3 Assessment of weight-adjusted-waist index

The WWI is a novel index that employs WC and weight to assess central obesity (15). Anthropometric assessments were conducted by skilled health technicians within a mobile examination center, with rigorous monitoring implemented through direct observation, thorough data reviews, and regular evaluations by expert examiners. The formula utilized for calculating the WWI involves dividing the waist circumference (measured in centimeters) by the square root of the weight (measured in kilograms). In our study, WWI was considered as a continuous variable and was designated as the exposure variable.

### 2.4 Covariates

Variables were chosen based on established knowledge and prior research as potential confounding factors. These included age (in years), sex (female, male), race/ethnicity background (Mexican American, Hispanic, Non-Hispanic White, Non-Hispanic Black, and Other), education level (under high school, high school or equivalent, and above high school), marital status (married, widowed, divorced, separated, never married and cohabiting), physical activity in leisure time (minutes/month), smoke status (never, former and current) and daily alcohol consumption (g/day). Physical activity in leisure time is defined as the time spent in moderate physical activity in a typical month. Excessive alcohol consumption is defined as >30 g/day for males and >20 g/day for females.

### 2.5 Statistical analysis

In the statistical analysis, the intricate design of the multistage cluster survey was duly considered, adhering strictly to the recommendations of the Centers for Disease Control and Prevention (CDC). Furthermore, suitable NHANES sampling weights were employed. For categorical variables, weighted descriptive statistics were presented in the form of unweighted numbers accompanied by weighted percentages, while continuous variables were depicted as the weighted mean ± standard error (SE).

Using multivariable logistic regression, we determined the prevalence of anxiety associated with WWI by calculating odds ratios (ORs) and their corresponding 95% confidence intervals (CIs). The lowest quartile of WWI served as the reference standard. Model 1 represents a crude model, which was not adjusted for any covariates, allowing us to assess the degree to which the selected covariates might confound these associations. Model 2 incorporated adjustments for age, sex, and race/ethnicity. Building upon Model 2, Model 3 further adjusted for education level, marital status, leisure-time physical activity, smoking status, and daily alcohol consumption. Subgroup analysis was conducted utilizing a stratified multivariable logistic regression model, incorporating stratified factors such as age, sex, race, educational attainment, marital status, smoking status, and heavydrink status.

To explore the potential non-linear relationship between WWI and anxiety symptoms, we employed generalized additive models (GAMs). Furthermore, we conducted stratifications based on age, sex, race, education level, marital status, leisure-time physical activity, and smoking status, respectively, to gain deeper insights into these associations.

To further evaluate the robustness of our primary findings, sensitivity analyses were conducted. Additionally, sleep duration and depressive state were adjusted as covariates to account for the influence of other social determinants. Moreover, the analysis incorporated the number of days of anxiety experienced per month (ranging from 0 to 30 days) and anxiolytic state into the multivariable linear/logistic regression model. Data analysis was performed utilizing Stata 17.0 and Empowerstats.^1^ Statistical significance was determined using a two-sided *P*-value threshold of <0.05.

## 3 Results

### 3.1 Characteristics of participants

Table 1 presents the sociodemographic characteristics of the participants according to their anxiety state. A total of 14677 individuals were included in this study. Significant differences were observed across the anxiety stratification groups in terms of age, sex, race, smoking status, physical activity level, marital status, educational attainment, and alcohol consumption (*P* < 0.05). Additionally, Supplementary Table 2 outlines the sociodemographic characteristics of individuals based on their anxiolytic state. The group reporting an anxiolytic state experienced a significantly higher number of anxious days per month compared to those without an anxiolytic state (*P* < 0.001).

**TABLE 1:** Characteristics of participants included in NHANES 2007–2012 analyses.

Variable	Total (*n* = 14677)	Anxiety state		*p*-value
		No (*n* = 10932)	Yes (*n* = 3745)	
Age (years)	47.05 ± 0.36	47.73 ± 0.38	45.10 ± 0.43	<0.001
Sex				<0.001
Female	7351 (50.87)	5123 (48.27)	2228 (58.40)	
Male	7326 (49.13)	5809 (51.73)	1517 (41.60)	
Race/ethnicity				0.007
Mexican American	2252 (8.10)	1703 (8.39)	549 (7.26)	
Other Hispanic	1540 (5.39)	1084 (5.08)	456 (6.25)	
Non-Hispanic White	6626 (69.13)	4823 (68.64)	1803 (70.55)	
Non-Hispanic Black	3098 (10.96)	2377 (11.08)	721 (10.61)	
Other Race	1161 (6.42)	945 (6.80)	216 (5.33)	
Smoking status				<0.001
Never	7913 (54.31)	6101 (56.38)	1812 (48.34)	
Ever	3595 (24.77)	2783 (25.56)	812 (22.50)	
Current	3169 (20.92)	2048 (18.06)	1121 (29.16)	
Physical activity in leisure time (minutes/month)	579.05 ± 16.32	593.66 ± 16.73	536.87 ± 27.38	0.039
Marital status				<0.001
Married	7535 (55.53)	5856 (57.57)	1679 (49.66)	
Widowed	1179 (5.43)	904 (5.66)	275 (4.76)	
Divorced	1610 (10.49)	1093 (9.51)	517 (13.31)	
Separated	503 (2.33)	324 (2.00)	179 (3.27)	
Never married	2699 (18.34)	1972 (18.08)	727 (19.10)	
Cohabiting	1151 (7.88)	783 (7.18)	368 (9.89)	
Education				<0.001
Under high school	3950 (17.93)	2816 (16.74)	1134 (21.36)	
High school or equivalent	3391 (22.79)	2583 (23.06)	808 (22.03)	
Above high school	7336 (59.28)	5533 (60.20)	1803 (56.61)	
Alcohol consumption (g/day)	7.33 ± 0.25	6.95 ± 0.25	8.44 ± 0.44	0.001

Continuous data were displayed as weighted mean ± standard error (SE), while categorical variables were exhibited as unweighted numbers (weighted percentages). *P* < 0.05 was regarded as statistically significant.

### 3.2 The association between WWI and anxiety

Logistic regression analysis revealed that, after accounting for all confounding factors, the correlation between WWI, and anxiety remained statistically significant. As illustrated in Table 2, a one-unit increase in WWI was associated with a 11% increase in the prevalence of anxiety. Additionally, a sensitivity analysis conducted using WWI as a categorical variable (quartile) further confirmed the significance of this association (OR = 1.25, 95% CI: 1.04–1.51; *P* = 0.021).

**TABLE 2:** Association between weight-adjusted-waist index (WWI) and anxiety.

OR (95%CI) *P*-value	Model 1	Model 2	Model 3
Continuous	1.05 (0.98, 1.12) 0.140	1.15 (1.06, 1.24) 0.001	1.11 (1.03, 1.20) 0.009
**WWI quartiles**			
Q1	Reference		
Q2	0.97 (0.84, 1.12) 0.684	1.07 (0.92, 1.24) 0.387	1.03 (0.88, 1.21) 0.674
Q3	0.97 (0.85, 1.11) 0.698	1.14 (0.97, 1.33) 0.101	1.09 (0.93, 1.28) 0.266
Q4	1.12 (0.96, 1.31) 0.162	1.33 (1.10, 1.62) 0.004	1.25 (1.04, 1.51) 0.021

OR, odds radio; CI, confidence interval. Model 1: no covariates were adjusted. Model 2: age, sex, and race/ethnicity were adjusted. Model 3: age, sex, race/ethnicity, education level, marital status, leisure-time physical activity, smoking status, and daily alcohol consumption were adjusted.

The relationships between the frequency of anxiety and WWI exhibited inconsistencies across the subgroups, as depicted in Table 3. The significant association between WWI and anxiety was observed among female, under 50 years old, non-Hispanic Black, separated, non-smokers, and non-heavy drinkers only. Furthermore, the subgroup analyses revealed that participants with an educational level below high school demonstrated strong associations between WWI and anxiety.

**TABLE 3:** Subgroup analysis of the association between weight-adjusted-waist index (WWI) and anxiety.

Subgroup	Model 1		Model 2		Model 3	
	OR (95%CI)	*P-*value	OR (95%CI)	*P-*value	OR (95%CI)	*P-*value
**Gender**						
Male	0.92 (0.84, 1.01)	0.075	1.08 (0.95, 1.23)	0.214	1.07 (0.95, 1.22)	0.268
Female	1.06 (0.97, 1.16)	0.195	1.18 (1.07, 1.31)	0.002	1.12 (1.01, 1.25)	0.037
Age						
<50	1.24 (1.14, 1.35)	<0.001	1.21 (1.10, 1.33)	<0.001	1.20 (1.10, 1.33)	<0.001
≥50	1.02 (0.88, 1.18)	0.811	0.97 (0.83, 1.13)	0.695	0.93 (0.80, 1.09)	0.372
**Race**						
Mexican American	1.27 (1.09, 1.49)	0.003	1.11 (0.93, 1.33)	0.251	1.09 (0.91, 1.31)	0.337
Other Hispanic	1.01 (0.86, 1.19)	0.868	0.92 (0.77, 1.11)	0.387	0.97 (0.81, 1.16)	0.718
Non-Hispanic White	1.00 (0.92, 1.09)	0.983	1.16 (1.05, 1.29)	0.006	1.11 (1.00, 1.23)	0.055
Non-Hispanic Black	1.20 (1.09, 1.33)	<0.001	1.16 (1.04, 1.30)	0.010	1.20 (1.06, 1.34)	0.004
Other Race	1.10 (0.88, 1.39)	0.394	1.22 (0.93, 1.59)	0.144	1.20 (0.90, 1.60)	0.207
**Education**						
Under high school	1.11 (0.97, 1.27)	0.120	1.16 (0.99, 1.35)	0.062	1.16 (1.00, 1.34)	0.047
High school or equivalent	1.01 (0.91, 1.12)	0.883	1.02 (0.88, 1.17)	0.824	1.01 (0.87, 1.17)	0.867
Above high school	1.00 (0.91, 1.10)	0.986	1.14 (1.01, 1.29)	0.032	1.14 (1.01, 1.29)	0.029
**Marital status**						
Married	1.06 (0.95, 1.19)	0.289	1.17 (1.04, 1.33)	0.013	1.13 (1.00, 1.29)	0.054
Widowed	0.94 (0.74, 1.19)	0.576	1.01 (0.78, 1.31)	0.919	0.99 (0.75, 1.29)	0.912
Divorced	1.10 (0.93, 1.30)	0.242	1.16 (0.96, 1.41)	0.119	1.12 (0.94, 1.35)	0.209
Separated	1.33 (0.98, 1.81)	0.068	1.62 (1.11, 2.37)	0.014	1.59 (1.06, 2.40)	0.027
Never married	1.16 (1.02, 1.32)	0.021	1.11 (0.95, 1.28)	0.173	1.11 (0.96, 1.29)	0.142
Living with partner	0.92 (0.76, 1.12)	0.414	0.97 (0.78, 1.21)	0.786	0.96 (0.76, 1.20)	0.700
**Smoking status**						
Never	1.04 (0.97, 1.12)	0.288	1.12 (1.01, 1.23)	0.028	1.12 (1.01, 1.24)	0.029
Ever	0.97 (0.85, 1.11)	0.614	1.09 (0.93, 1.29)	0.270	1.06 (0.91, 1.25)	0.439
Current	1.21 (1.08, 1.36)	0.001	1.18 (1.02, 1.35)	0.024	1.15 (1.00, 1.33)	0.051
**Heavydrink**						
Yes	0.99 (0.81, 1.21)	0.914	0.90 (0.64, 1.27)	0.557	0.9 (0.66, 1.31)	0.678
No	1.06 (1.00, 1.13)	0.068	1.16 (1.07, 1.26)	<0.001	1.1 (1.03, 1.21)	0.008

OR, odds radio; CI, confidence interval. Model 1: no covariates were adjusted. Model 2: age, sex, and race/ethnicity were adjusted. Model 3: age, sex, race/ethnicity, education level, marital status, leisure-time physical activity, smoking status, and daily alcohol consumption were adjusted.

### 3.3 A non-linear relationship between WWI and anxiety

**FIGURE 2:**
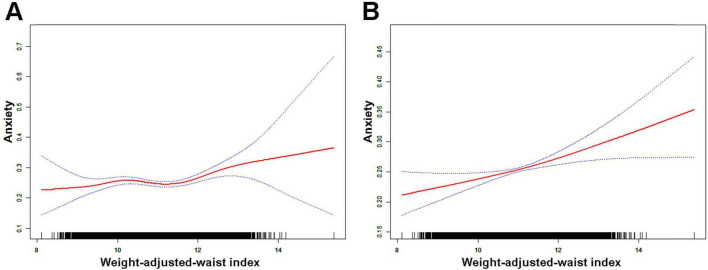
Results of smoothed curve fitting between weight-adjusted-waist index and anxiety. **(A)** Unadjusted for variables; **(B)** adjusted for age, sex, race/ethnicity, education level, marital status, leisure-time physical activity, smoking status, and daily alcohol consumption. The red solid arcs indicate the smoothed curve fitting between the variables. The area between the two blue dashed lines represents the 95% CI.

**FIGURE 3:**
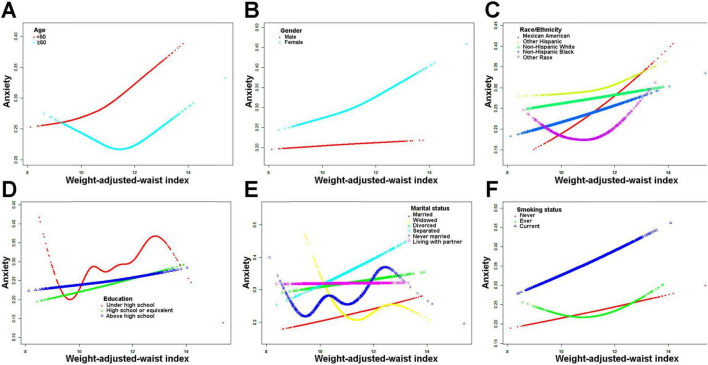
Results of smoothed curve fitting between weight-adjusted-waist index and anxiety stratified by age, sex, race, education level, marital status, leisure-time physical activity, and smoking status. **(A)** Stratified by age; **(B)** Stratified by gender; **(C)** Stratified by race; **(D)** Stratified by education level; **(E)** Stratified by marital status; **(F)** Stratified by smoking status.

Figure 2 depicts the non-linear correlation between WWI and anxiety, which was derived from the generalized additive model analysis. Figure 2A presents the unadjusted data, demonstrating that anxiety risk increases as WWI levels increase. Figure 2B displays the data after adjusting for all variables, showing a similar trend where anxiety risk increases with increasing WWI levels. Figure 3 showcases the results of the smoothed curve fit, revealing an overall increasing trend in anxiety risk with rising WWI levels after stratifying by various factors, including age, sex, race, educational attainment, marital status, and smoking status.

### 3.4 Sensitivity analysis

As illustrated in Supplementary Tables 3, 4, the associations observed between WWI and the risk of anxiety (assessed by the number of anxiety days or the use of anxiolytics as primary outcomes) were highly consistent with our primary findings.

Moreover, even after adjusting for additional social factors like sleep duration (Supplementary Table 5), the associations between WWI with anxiety risk remained consistent. Nevertheless, when additional adjustments were made for social factors such as depressive state (Supplementary Table 5), the relationships between WWI and the risk of anxiety were found to be insignificant. This led us to investigate the potential interaction between WWI and depressive states regarding the risk of anxiety, which resulted in a significant finding (P for interaction: 0.725) (Supplementary Table 6).

## 4 Discussion

To our current knowledge, this study marks the inaugural evaluation of the associations between WWI and anxiety risk within a broad public context. Leveraging a large, nationally representative sample from NHANES, our findings were designed to enhance their general applicability. In our study, which enrolled a total of 14,677 participants, we uncovered a robust positive relationship between the WWI and the severity of anxiety symptoms. This positive association remained consistent even when WWI was stratified into quartiles (Q1-Q4). Furthermore, through subgroup analyses, we observed that the association was more pronounced among individuals who were female, under 50 years old, non-Hispanic Black, separated, non-smokers, and non-heavy drinkers. Given the intricate nature of the development and recurrence of anxiety symptoms, understanding the associated risk factors is paramount for devising effective prevention and treatment strategies. Our findings highlight the significant clinical utility of WWI in identifying anxietysymptoms and promoting early disease detection. Moreover, the identification of pertinent biomarkers continues to be crucial for the timely recognition of anxiety symptoms in individuals.

While the dataset lacked specific anxiety questionnaires such as the State-Trait Anxiety Inventory (STAI) or the Beck Anxiety Inventory-Primary Care (BAI-PC), we ensured a comprehensive assessment of anxiety burden by engaging trained investigators to inquire about the participants’ recent frequency of anxiety experiences. In line with previous studies, we defined high anxiety levels as experiencing anxiety for 7 or more days within the past 30 days, a criterion we incorporated into our analysis. Additionally, we employed anxious days as a continuous outcome variable to enrich our analysis. Furthermore, we explored the relationships between WWI and the use of anxiolytics, thus allowing us to conduct a thorough investigation into the correlation between WWI and anxiety risk from multiple perspectives.

The relationship between WWI and anxiety exhibits variations across demographic and lifestyle factors. Age differences may stem from age-related declines in muscle synthesis capabilities and the prevalence of chronic health issues among older individuals (29). Education level could be indicative of socioeconomic status and access to resources, whereas marital status might be associated with social support, familial stress, and overall life stability (30). Disparities in smoking and drinking habits could be attributed to the impacts of these behaviors on fat metabolism, the immune system, stress-coping mechanisms, and social engagement. Further research is imperative to validate these findings and elucidate the underlying reasons for such discrepancies.

The mechanisms underlying the distinct relationships between depression and obesity exhibited by women and men remain unknown. One plausible explanation could be that this sex-specific association between obesity and anxiety underscores sex differences in biological susceptibility and may also be intrinsically tied to sex hormones, which have been reported to be associated with anxiety (31, 32). Additionally, one factor contributing to increased anxiety among women with overweight and obesity may be the stigma associated with weight and the more severe discrimination faced by women (33). The inherently greater dissatisfaction with their bodies among females, coupled with societal pressures to maintain a thin physique, can impact self-esteem and elevate stress levels. This, in turn, may heighten the risk of both obesity and anxiety, particularly among females throughout their lifespan. Although obesogenic foods (e.g., high carbohydrates) may possess mood-enhancing qualities, the stigma attached to obesity for women may counteract these benefits, leading to an elevated risk of anxiety (34, 35). Overweight women are more prone to dissatisfaction with their weight and encounter social challenges compared to women of a desirable weight (33, 36).

Our study found that the association between WWI and anxiety was only present in non-Hispanic Black people. Regarding racial differences, there may be genetic disparities as well as variations in social, cultural, and economic factors. However, several report that White, but not Black or Hispanic women, showed a positive association between obesity and mood disorders (37, 38). Multiple lines of evidence indicate that racial and ethnic groups exhibit variations in their ideals of bodily appearance (34). Moreover, the contextual disparities between Black and White individuals, encompassing both coping resources and constraints as well as societal norms surrounding body shape, contribute to distinct stress-related outcomes (34). There is a need for further investigation to attain a clear and comprehensive understanding of this relationship.

Currently, the majority of research investigating the connection between obesity and depression relies on basic obesity indicators, such as BMI and waist circumference, with only a limited number of studies utilizing the WWI to assess central obesity (39). While most earlier research has relied on BMI to gauge the connection between obesity and anxiety, this approach has faced consistent criticism. BMI primarily emphasizes overall weight, neglecting to adequately differentiate between various tissue compositions (such as adipose, muscle, and bone) or the distribution of adipose tissue (whether in the upper or lower body). This oversight can potentially skew estimates (40, 41). WC is regarded as an alternative indicator for indirectly evaluating visceral fat accumulation (42). However, akin to BMI, WC alone is unable to differentiate between visceral fat and subcutaneous fat (13). To rectify this limitation, our study employed the Waist-to-Weight Index (WWI) to explore the relationship between obesity and anxiety. Initially introduced to reflect abdominal tissue composition, WWI exhibits a notable positive correlation with abdominal fat mass and a negative correlation with abdominal muscle mass (43, 44). Our study revealed a non-linear, positive association between WWI and anxiety, potentially attributed to distinct patterns of fat distribution. Beyond fat and muscle mass, recent research has reported positive correlations between WWI and an elevated risk of both osteoporosis and fractures (45, 46). Notably, a previous study highlighted that complications associated with osteoporosis significantly impact individuals’ lives and often result in anxiety (47). Consequently, WWI has recently been recognized as a comprehensive index that reflects unfavorable body compositions, encompassing abdominal obesity, muscle loss, and reduced bone mineral density (18).

The WWI represents a newly devised obesity indicator, exhibiting enhanced accuracy in evaluating central obesity and has been investigated across various domains. Since its inception as a biomarker in 2018, the diagnostic and prognostic utility of the WWI has been primarily established in clinical trials focused on liver and cardiovascular diseases (16, 48). Recent findings indicate that WWI is emerging as a straightforward and effective marker for predicting and diagnosing renal function (20), orthopedic conditions (46), respiratory illnesses (44), and more. Notably, WWI also holds implications for dementia, demonstrating an independent and positive correlation with dementia in hypertensive individuals, and serving as a practical tool for assessing dementia risk in clinical settings. However, there are no studies related to anxiety, warranting increased attention.

In contrast to the measurement of visceral fat, which necessitates expensive and resource-intensive equipment such as MRI, WWI offers an easily accessible and cost-effective obesity index, particularly suitable for less developed regions. WWI remains relatively unaffected by factors such as BMI changes, thus providing a more accurate reflection of dementia risk in clinical practice (15, 44). Additionally, WWI exhibits a linear and positive correlation with cardiometabolic morbidity and mortality, demonstrating excellent predictive capabilities for cardiometabolic and cardiovascular diseases (49, 50). Furthermore, WWI serves as a simple anthropometric index for effectively predicting albuminuria, outperforming other obesity indicators in terms of correlation (20, 50). As an anthropometric indicator, the WWI is anticipated to be further explored due to its straightforward calculation and exceptional performance in assessing disease. In conclusion, there is widespread belief that the WWI can serve as a predictive marker for obesity-related diseases, and our research provides evidence to support this notion.

The unhealthy lifestyle habits commonly adopted by obese individuals, including inadequate exercise and excessive eating, can exert a detrimental impact on their mental well-being (51). Conversely, anxiety may also foster the development of unhealthy lifestyle patterns, thereby creating a vicious cycle that exacerbates other chronic conditions. Moreover, the interplay between obesity and anxiety is grounded in both biological and socio-psychological factors. From a biological standpoint, obesity appears to play a pivotal role in both the initiation and progression of diabetes and insulin resistance. Additionally, the subtle inflammatory processes triggered by various diseases may contribute to the development of anxiety (52). Furthermore, obesity can impair the proper functioning of adipose tissue, leading to significant ectopic accumulation that disrupts normal physiological processes and exacerbates the onset and progression of metabolic-related diseases (53, 54). Notably, central obesity stands out as a prime example of “dysfunctional adipose tissue,” posing the highest health risks due to its ectopic accumulation (55). This type of obesity may also facilitate the emergence and progression of anxiety through neuroendocrine disruptions within the hypothalamic-pituitary-adrenal axis (56).

Being overweight and having an irregular distribution of white fat are associated with an elevated WWI. An excessive amount of white adipose tissue, particularly when abnormally distributed in the viscera, can prompt immune cells to overexpress Lipopolysaccharide (LPS) and Toll-Like Receptor 4 (TLR4) (57). This overexpression triggers an inflammatory cascade, leading to the release of various inflammatory factors, including TNF-α, IL-1β, IL-6, MCP1, and CRP (57). These factors contribute to oxidative stress and insulin resistance. When these factors converge, neuronal apoptosis ensues (58). This process disrupts the mitochondrial activity of neurons within the central nervous system, particularly in the hippocampus and frontotemporal lobe—two primary brain regions crucial for regulating emotions and mental processes in humans (8). Ultimately, this disruption results in anxiety.

In addition to biological mechanisms, it is crucial to also consider social psychological factors. The perception of being overweight or obese can heighten psychological distress, foster dissatisfaction with personal body image, and undermine self-esteem (51). The accumulation of psychological stress and shame associated with weight in obese individuals can elevate the risk of developing anxiety. Individuals with obesity often encounter weight stigma and discrimination throughout their entire lives, leading to enduring psychological distress (59). Regarding psychosocial processes, individuals with a higher WWI tend to encounter greater social stress and challenges concerning their self-image, thereby potentially increasing their vulnerability to anxiety (8). Moreover, emotional eating, a phenomenon where individuals consume food as an emotional response to negative arousal, has been demonstrated to be associated with weight gain (33).

In our study, we observed a link between WWI and an elevated risk of anxiety. However, after additional adjustment for covariates such as depressive state, this association was attenuated. Our study found that there is no potential interaction between antimony exposure and the co-occurrence of depression and anxiety. Therefore, while the individual relationships between WWI and anxiety, as well as WWI and depression, are evident, the idea that WWI is more likely to elevate the risk of both depression and anxiety concurrently is not supported by our current findings. Nevertheless, given the complexity of these relationships and the potential influence of various factors, further research is still needed to comprehensively understand the associations between WWI, depression, and anxiety.

Our study offers several advantages. Firstly, to ensure the credibility of our findings, we employed appropriate weighting and made confounding adjustments during our analyses. Secondly, our sample size was sufficiently large to effectively uncover the relationship between WWI and anxiety. To enhance the reliability of our results, we conducted sensitivity analyses on a substantial sample size and made concerted efforts to take into account various factors that could potentially influence the outcomes. Lastly, we specifically concentrated on exploring the link between WWI levels and anxiety. In contrast, relatively few studies have delved into this relationship, with most research focusing on the connection between WWI and depression.

Inevitably, our study also has several limitations. First and foremost, owing to the intrinsic nature of cross-sectional study designs, limiting our ability to thoroughly investigate causal relationships. A considerable number of clinical cohort studies are essential to verify and confirm our conclusions. Secondly, while the dependent variable exhibited a high level of confidence, it’s important to note that the measurements were derived from questionnaires, which may be susceptible to the subjectivity of the respondents. Furthermore, while we adjusted for certain covariates, it was not possible to account for all potential factors, and uncontrolled variables could still impact our conclusions. Finally, although the NHANES cohort comprised participants from various ethnic and socioeconomic backgrounds, it is important to note that all participants were from the United States. Consequently, our findings necessitate validation in populations from other countries, particularly those in low-to-middle-income nations. Additionally, we used the 2007 - 2012 NHANES data, which is old and may have biases due to changes in mental health data collection and reporting over time. We chose this dataset based on other studies’ practices and its unique anxiety - related data, but more recent data would be better.

## 5 Conclusion

Through the analysis of a nationally representative sample, our study has uncovered a notable association between a high WWI and an elevated number of anxiety symptoms. Regarding national health care, it is imperative to focus on weight control and modifying abnormal body fat distribution in order to decrease the occurrence of anxiety. Given the complexity of this relationship, further research is required to validate and delve into the underlying mechanisms.

## Data Availability

Publicly available datasets were analyzed in this study. This data can be found here: https://www.cdc.gov/nchs/nhanes/index.htm.

## Footnotes

1
http://www.empowerstats.com
