# Supplementary material for: Association between weight-adjusted-waist index and anxiety among adults in the National Health and Nutrition Examination Survey (NHANES), 2007–2012
Source: Front Nutr. 2025 Jun 13;12:1530028. doi: 10.3389/fnut.2025.1530028 (PMC12202602; doi:10.3389/fnut.2025.1530028)
Supplement: Supplementary file 1 [file Data_Sheet_1.docx]

**Table S1.** **The codes and names of drugs** **utilized in defining the anxiolytic state.**

| **Rxddrgid** | **Drug name** | **Therapeutic category** |
| --- | --- | --- |
| d03456 | ACETAMINOPHEN; BUTALBITAL | central nervous system agents; analgesics; miscellaneous analgesics; anxiolytics, sedatives, and hypnotics; barbiturates |
| d03455 | ACETAMINOPHEN; BUTALBITAL; CAFFEINE | central nervous system agents; analgesics; miscellaneous analgesics; anxiolytics, sedatives, and hypnotics; barbiturates; cns stimulants |
| d03425 | ACETAMINOPHEN; BUTALBITAL; CAFFEINE; CODEINE | central nervous system agents; analgesics; miscellaneous analgesics; narcotic analgesics; anxiolytics, sedatives, and hypnotics; barbiturates; cns stimulants; respiratory agents; antitussives |
| d03459 | ACETAMINOPHEN; DICHLORALPHENAZONE; ISOMETHEPTENE | central nervous system agents; analgesics; miscellaneous analgesics; antimigraine agents; anxiolytics, sedatives, and hypnotics; miscellaneous anxiolytics, sedatives and hypnotics |
| d00168 | ALPRAZOLAM | central nervous system agents; anxiolytics, sedatives, and hypnotics; benzodiazepines |
| d03462 | AMITRIPTYLINE; CHLORDIAZEPOXIDE | central nervous system agents; anxiolytics, sedatives, and hypnotics; benzodiazepines; psychotherapeutic agents; antidepressants; tricyclic antidepressants |
| c00067 | ANXIOLYTICS, SEDATIVES, AND HYPNOTICS - UNSPECIFIED | central nervous system agents; anxiolytics, sedatives, and hypnotics |
| d03458 | ASPIRIN; BUTALBITAL | central nervous system agents; analgesics; salicylates; anxiolytics, sedatives, and hypnotics; barbiturates; coagulation modifiers; antiplatelet agents; platelet aggregation inhibitors |
| d03457 | ASPIRIN; BUTALBITAL; CAFFEINE | central nervous system agents; analgesics; salicylates; anxiolytics, sedatives, and hypnotics; barbiturates; cns stimulants; coagulation modifiers; antiplatelet agents; platelet aggregation inhibitors |
| d03426 | ASPIRIN; BUTALBITAL; CAFFEINE; CODEINE | central nervous system agents; analgesics; narcotic analgesics; salicylates; anxiolytics, sedatives, and hypnotics; barbiturates; cns stimulants; coagulation modifiers; antiplatelet agents; platelet aggregation inhibitors; respiratory agents; antitussives |
| d03485 | ATROPINE; HYOSCYAMINE; PHENOBARBITAL; SCOPOLAMINE | cardiovascular agents; anticholinergic chronotropic agents; central nervous system agents; anticonvulsants; barbiturate anticonvulsants; antiemetic/antivertigo agents; anticholinergic antiemetics; anxiolytics, sedatives, and hypnotics; barbiturates; gastrointestinal agents; functional bowel disorder agents; anticholinergics/antispasmodics; miscellaneous agents; antidotes |
| d03495 | BELLADONNA; ERGOTAMINE; PHENOBARBITAL | central nervous system agents; analgesics; antimigraine agents; anticonvulsants; barbiturate anticonvulsants; anxiolytics, sedatives, and hypnotics; barbiturates; gastrointestinal agents; functional bowel disorder agents; anticholinergics/antispasmodics |
| d07064 | BROMPHENIRAMINE; DIPHENHYDRAMINE; PHENYLEPHRINE | cardiovascular agents; vasopressors; central nervous system agents; antiemetic antivertigo agents; anticholinergic antiemetics; antiparkinson agents; anticholinergic antiparkinson agents; anxiolytics, sedatives, and hypnotics; miscellaneous anxiolytics, sedatives and hypnotics; respiratory agents; antihistamines; decongestants |
| d00182 | BUSPIRONE | central nervous system agents; anxiolytics, sedatives, and hypnotics; miscellaneous anxiolytics, sedatives and hypnotics |
| d00923 | BUTABARBITAL | central nervous system agents; anxiolytics, sedatives, and hypnotics; barbiturates |
| d03061 | BUTALBITAL | central nervous system agents; anxiolytics, sedatives, and hypnotics; barbiturates |
| d07351 | CARBETAPENTANE; PSEUDOEPHEDRINE; PYRILAMINE | central nervous system agents; anxiolytics, sedatives, and hypnotics; miscellaneous anxiolytics, sedatives and hypnotics; respiratory agents; antihistamines; antitussives; decongestants |
| d00147 | CHLORAL HYDRATE | central nervous system agents; anxiolytics, sedatives, and hypnotics; miscellaneous anxiolytics, sedatives and hypnotics |
| d03492 | CHLORDIAZEPOXIDE; CLIDINIUM | central nervous system agents; anxiolytics, sedatives, and hypnotics; benzodiazepines; gastrointestinal agents; functional bowel disorder agents; anticholinergics/antispasmodics |
| d03313 | CHLORPHENIRAMINE; PHENYLEPHRINE; PYRILAMINE | cardiovascular agents; vasopressors; central nervous system agents; anxiolytics, sedatives, and hypnotics; miscellaneous anxiolytics, sedatives and hypnotics; respiratory agents; antihistamines; decongestants |
| d00198 | CLORAZEPATE | central nervous system agents; anxiolytics, sedatives, and hypnotics; benzodiazepines |
| d03371 | DEXTROMETHORPHAN; PHENYLEPHRINE; PYRILAMINE | cardiovascular agents; vasopressors; central nervous system agents; anxiolytics, sedatives, and hypnotics; miscellaneous anxiolytics, sedatives and hypnotics; respiratory agents; antihistamines; antitussives; decongestants |
| d00148 | DIAZEPAM | central nervous system agents; anticonvulsants; benzodiazepine anticonvulsants; anxiolytics, sedatives, and hypnotics; benzodiazepines |
| d00212 | DIPHENHYDRAMINE | central nervous system agents; antiemetic/antivertigo agents; anticholinergic antiemetics; antiparkinson agents; anticholinergic antiparkinson agents; anxiolytics, sedatives, and hypnotics; miscellaneous anxiolytics, sedatives and hypnotics; respiratory agents; antihistamines |
| d00217 | DOXEPIN | central nervous system agents; anxiolytics, sedatives, and hypnotics; miscellaneous anxiolytics, sedatives and hypnotics; psychotherapeutic agents; antidepressants; tricyclic antidepressants |
| d00915 | ESTAZOLAM | central nervous system agents; anxiolytics, sedatives, and hypnotics; benzodiazepines |
| d05421 | ESZOPICLONE | central nervous system agents; anxiolytics, sedatives, and hypnotics; miscellaneous anxiolytics, sedatives and hypnotics |
| d00238 | FLURAZEPAM | central nervous system agents; anxiolytics, sedatives, and hypnotics; benzodiazepines |
| d04903 | GUAIFENESIN; PHENYLEPHRINE; PYRILAMINE | cardiovascular agents; vasopressors; central nervous system agents; anxiolytics, sedatives, and hypnotics; miscellaneous anxiolytics, sedatives and hypnotics; respiratory agents; antihistamines; decongestants; expectorants |
| d00907 | HYDROXYZINE | central nervous system agents; anxiolytics, sedatives, and hypnotics; miscellaneous anxiolytics, sedatives and hypnotics; respiratory agents; antihistamines |
| d00149 | LORAZEPAM | central nervous system agents; anticonvulsants; benzodiazepine anticonvulsants; antiemetic/antivertigo agents; miscellaneous antiemetics; anxiolytics, sedatives, and hypnotics; benzodiazepines |
| d04058 | MELATONIN | central nervous system agents; anxiolytics, sedatives, and hypnotics; miscellaneous anxiolytics, sedatives and hypnotics; alternative medicines; nutraceutical products |
| d00040 | OXAZEPAM | central nervous system agents; anxiolytics, sedatives, and hypnotics; benzodiazepines |
| d00340 | PHENOBARBITAL | central nervous system agents; anticonvulsants; barbiturate anticonvulsants; anxiolytics, sedatives, and hypnotics; barbiturates |
| d04446 | PHENYLEPHRINE; PYRILAMINE | cardiovascular agents; vasopressors; central nervous system agents; anxiolytics, sedatives, and hypnotics; miscellaneous anxiolytics, sedatives and hypnotics; respiratory agents; antihistamines; decongestants |
| d05578 | RAMELTEON | central nervous system agents; anxiolytics, sedatives, and hypnotics; miscellaneous anxiolytics, sedatives and hypnotics |
| d00384 | TEMAZEPAM | central nervous system agents; anxiolytics, sedatives, and hypnotics; benzodiazepines |
| d00397 | TRIAZOLAM | central nervous system agents; anxiolytics, sedatives, and hypnotics; benzodiazepines |
| d04452 | ZALEPLON | central nervous system agents; anxiolytics, sedatives, and hypnotics; miscellaneous anxiolytics, sedatives and hypnotics |
| d00910 | ZOLPIDEM | central nervous system agents; anxiolytics, sedatives, and hypnotics; miscellaneous anxiolytics, sedatives and hypnotics |

**Table S2. Characteristics of participants included in NHANES 2007-2012 analyses categorized by anxiolytics state.**

| **Variable** | **Total(n=14677)** | **Anxiolytics** | | ***p* value** |
| --- | --- | --- | --- | --- |
|  |  | **No(n=10397)** | **Yes(n=4280)** |  |
| **Age(years)** | 47.05 ± 0.36 | 47.72 ± 0.37 | 45.53 ± 0.47 | <0.001 |
| **Sex** |  |  |  | <0.001 |
| Female | 7351(50.87) | 4699(45.80) | 2652(62.48) |  |
| Male | 7326(49.13) | 5698(54.20) | 1629(37.52) |  |
| **Race/ethnicity** |  |  |  | <0.001 |
| Mexican American | 2252(8.10) | 1666(8.78) | 586(6.56) |  |
| Other Hispanic | 1540(5.39) | 1056(5.30) | 484(5.57) |  |
| Non-Hispanic White | 6626(69.13) | 4469(67.41) | 2157(73.07) |  |
| Non-Hispanic Black | 3098(10.96) | 2289(11.42) | 809(9.91) |  |
| Other Race | 1161(6.42) | 917(7.09) | 244(4.89) |  |
| **Smoking status** |  |  |  | <0.001 |
| Never | 7913(54.31) | 5789(56.24) | 2124(49.92) |  |
| Ever | 3595(24.77) | 2624(25.41) | 971(23.30) |  |
| Current | 3169(20.92) | 1984(18.35) | 1185(26.78) |  |
| **Physical activity in leisure time(minutes/month)** | 579.05 ± 16.32 | 592.94 ± 16.29 | 547.31 ± 25.49 | 0.058 |
| **Marital status** |  |  |  | <0.001 |
| Married | 7535(55.53) | 5569(57.62) | 1966(50.77) |  |
| Widowed | 1179(5.43) | 844(5.53) | 335(5.20) |  |
| divorced | 1610(10.49) | 1038(9.53) | 572(12.68) |  |
| separated | 503(2.33) | 310(1.98) | 193(3.12) |  |
| Never married | 2699(18.34) | 1878(18.06) | 821(19.00) |  |
| cohabiting | 1151(7.88) | 758(7.28) | 393(9.24) |  |
| **Education** |  |  |  | 0.003 |
| Under high school | 3950(17.93) | 2734(17.24) | 1216(19.52) |  |
| High school or equivalent | 3391(22.79) | 2480(23.44) | 911(21.30) |  |
| Above high school | 7336(59.28) | 5183(59.32) | 2153(59.18) |  |
| **Alcohol consumption (g/day)** | 7.33 ± 0.25 | 7.08 ± 0.26 | 7.91 ± 0.39 | 0.033 |
| **Anxious days (0-30 days per month)** | 5.85 ± 0.12 | 1.22 ± 0.31 | 16.42 ± 0.25 | <0.001 |

Continuous data were displayed as weighted mean ± standard error (SE), while categorical variables were exhibited as unweighted numbers (weighted percentages).

*P* < 0.05 was regarded as statistically significant..

**Table S3. Association between weight-adjusted-waist index (WWI) and anxious days.**

| **β(95%CI)  *P* value** | **Model 1** | **Model 2** | **Model 3** |
| --- | --- | --- | --- |
| Continuous | 0.26(0.01,0.52) 0.039 | 0.61(0.28,0.93) <0.001 | 0.45(0.14,0.76) 0.005 |
| WWI quartiles | | | |
| Q1 | Reference | | |
| Q2 | -0.20(-0.68,0.27) 0.399 | 1.16(-0.34,0.66) 0.519 | 0.01(-0.49,0.52) 0.960 |
| Q3 | 0.14(-0.39,0.66) 0.607 | 0.73(0.10,1.36) 0.023 | 0.55(-0.05,1.14) 0.070 |
| Q4 | 0.57(-0.04,1.19) 0.068 | 1.24(0.49,1.99) 0.002 | 0.92(0.20,1.63) 0.013 |

Abbreviations: β, beta; CI, confidence interval.

Model 1: no covariates were adjusted.

Model 2: age, sex, and race/ethnicity were adjusted.

Model 3: age, sex, race/ethnicity, education level, marital status, leisure-time physical activity, smoking status, and daily alcohol consumption were adjusted.

**Table S4. Association between weight-adjusted-waist index (WWI) and anxiolytic state.**

| **OR(95%CI)  *P* value** | **Model 1** | **Model 2** | **Model 3** |
| --- | --- | --- | --- |
| Continuous | 1.04(0.98,1.10) 0.148 | 1.09(1.02,1.17) 0.015 | 1.07(1.00,1.14) 0.038 |
| WWI quartiles | | | |
| Q1 | Reference | | |
| Q2 | 0.95(0.83,1.08) 0.435 | 1.02(0.89,1.16) 0.770 | 1.00(0.88,1.14) 0.995 |
| Q3 | 0.97(0.86,1.10) 0.662 | 1.09(0.95,1.24) 0.200 | 1.06(0.93,1.22) 0.347 |
| Q4 | 1.10(0.96,1.25) 0.184 | 1.20(1.02,1.42) 0.031 | 1.15(0.99,1.34) 0.062 |

Abbreviations: OR, Odds radio; CI, confidence interval.

Model 1: no covariates were adjusted.

Model 2: age, sex, and race/ethnicity were adjusted.

Model 3: age, sex, race/ethnicity, education level, marital status, leisure-time physical activity, smoking status, and daily alcohol consumption were adjusted.

**Table S5. The associations between weight-adjusted-waist index (WWI) and odds ratios for anxiety.**

| **OR(95%CI)  *P* value** | **Model 4** | **Model 5** | **Model 6** |
| --- | --- | --- | --- |
| Continuous | 1.10(1.02,1.19) 0.016 | 1.03(0.95,1.13) 0.434 | 1.03(0.94,1.12) 0.503 |
| WWI quartiles | | | |
| Q1 | Reference | |  |
| Q2 | 1.03(0.87,1.21) 0.722 | 0.99(0.83,1.19) 0.951 | 0.99(0.82,1.19)  0.934 |
| Q3 | 1.08(0.92,1.27) 0.344 | 1.02(0.84,1.22) 0.871 | 1.01(0.83,1.21) 0.951 |
| Q4 | 1.23(1.02,1.49) 0.028 | 1.07(0.87,1.31) 0.521 | 1.06(0.86,1.31) 0.560 |

Abbreviations: OR, Odds radio; CI, confidence interval.

Model 4: age, sex, race/ethnicity, education level, marital status, leisure-time physical activity, smoking status, daily alcohol consumption , and sleep duration were adjusted.

Model 5: age, sex, race/ethnicity, education level, marital status, leisure-time physical activity, smoking status, daily alcohol consumption , and depression state (PHQ-9 scores < 10 or PHQ-9 scores ≥ 10) were adjusted.

Model 6: age, sex, race/ethnicity, education level, marital status, leisure-time physical activity, smoking status, daily alcohol consumption , sleep duration ,and depression state (PHQ-9 scores < 10 or PHQ-9 scores ≥ 10) were adjusted.

**Table S6. Interaction of weight-adjusted-waist index (WWI) and depression state on anxiety.**

| **Variable** | **OR** | ***P* for trend** | ***P* for interaction** |
| --- | --- | --- | --- |
| Continuous WWI | 1.41(1.25,1.59) | <0.001 | 0.725 |
| WWI quartiles |  |  |  |
| Q1 | Reference | |  |
| Q2 | 1.20(0.96,1.51) | 0.113 |  |
| Q3 | 1.51(1.17,1.93) | 0.002 |  |
| Q4 | 2.07(1.55,2.77) | <0.001 |  |

Abbreviations: OR, Odds radio; CI, confidence interval.

Model was adjusted for age, sex, race/ethnicity, education level, marital status, leisure-time physical activity, smoking status, and daily alcohol consumption.
